# Palliative Radiotherapy in Non-small Cell Lung Cancer: Patterns of Use and Predictors of 30-Day Mortality in End-of-Life Care

**DOI:** 10.7759/cureus.65238

**Published:** 2024-07-24

**Authors:** Dave Tik Fung Liu, Rahul Misra, Thomas Moore

**Affiliations:** 1 Oncology, Kent Oncology Centre, Kent, GBR

**Keywords:** single-fraction radiotherapy, non-small cell lung carcinoma (nsclc), radiation oncoolgy, end-of-life decision-making, palliative radiation therapy

## Abstract

Introduction

Lung cancer is the leading cause of cancer-related deaths worldwide, with non-small cell lung cancer (NSCLC) being the most common type. More than half of patients require radiotherapy throughout their treatment. Palliative radiotherapy (PRT) is an important tool for symptom control and quality of life improvement in advanced NSCLC patients. However, the benefits of PRT must be balanced against possible disadvantages, especially in end-of-life (EOL) care. This study aims to describe the profile of PRT-treated deceased NSCLC patients, quantify the proportion of PRT recipients in the last 30 days of life and identify short-term survival prognostic factors in this group.

Materials and methods

This retrospective analysis was performed at two radiotherapy facilities within the Kent Oncology Centre, UK, for two years, running from January 1, 2022, to January 1, 2024. Data were collected from 857 deceased NSCLC patients who received PRT. Demographics, cancer diagnosis, histology, tumour, node, metastasis (TNM) staging, radiotherapy details, recent treatments, performance status (PS) and comorbidities were analysed. Patients have been stratified as long-term survivors (more than 30 days after PRT initiation, LTS group) along with short-term survivors (STS) (died within 30 days, STS group). Descriptive statistics, chi-squared tests, t-tests and multivariable logistic regression have been used in the data analysis.

Results

Out of 857 patients, 148 (17.3%) died within 30 days of PRT initiation. PS was considerably worse (p = 0.027), Adult Comorbidity Evaluation 27 (ACE-27) scores were higher (p = 0.018), and metastatic disease was more prevalent (60.1% vs. 47.5%, p = 0.02) in STS group patients. Fewer patients in the STS group completed their treatment compared to the LTS group (63.5% vs. 82.8%, p < 0.001). The STS group also received lower mean radiation dose (17.7 Gy vs. 19.6 Gy, p = 0.022) and fewer fractions (4.4 vs. 5.2, p = 0.019). The most common RT regimen in both cohorts was 20 Gy in five fractions, used in 55.4% of STS and 49.8% of LTS patients, with no significant difference in single fraction RT use between groups (33.1% in STS vs. 36.8% in LTS, p = 0.401). Multivariate logistic regression identified significant predictors of 30-day mortality: poorer PS (adjusted OR: 1.981, 95% CI: 1.33-3.12, p = 0.001), metastatic disease (adjusted OR: 2.02, 95% CI: 1.246-3.571, p = 0.002), incomplete PRT (adjusted OR: 0.337, 95% CI: 0.21-0.514, p < 0.001) and no recent chemotherapy (adjusted OR: 0.542, 95% CI: 0.342-0.941, p = 0.044).

Conclusion

This study demonstrated that compared with previous reports, a higher proportion of NSCLC patients who received PRT died within 30 days of treatment initiation, and low treatment adherence rates highlight challenges in EOL settings. Identification of poor PS and metastatic disease as predictors of short-term mortality would help inform PRT decision-making. The underutilisation of single-fraction radiotherapy and the link between recent chemotherapy and lower 30-day mortality warrant further study. These results highlight the need for better prognostic tools and more selective use of PRT, including increased consideration of single-fraction radiotherapy, in NSCLC patients approaching end of life and emphasise the importance of balancing benefit against treatment burden in this vulnerable population.

## Introduction

Non-small cell lung cancer (NSCLC) is the most common type of lung cancer, while lung cancer remains the leading cause of cancer-associated deaths around the world [1]. Over half of all NSCLC patients require radiotherapy (RT) for local or metastatic disease [2], and most receive RT for palliative reasons [3]. Palliative radiotherapy (PRT) is an important tool for symptom management and enhancing quality of life in advanced NSCLC patients.

PRT has been proven effective in various clinical settings. In patients with brain or spinal cord metastases, it can improve neurological function and prevent further degeneration [4]. PRT has a good response rate of about 60% [5] for bone metastases and usually produces stable or improved neurologic symptoms in about half of brain metastases [6].

However, the benefits of PRT must be balanced against possible disadvantages, especially in end-of-life (EOL) care. While PRT for bone metastases typically takes weeks to result in symptomatic improvement [7], PRT for brain metastases has side effects and may not improve overall survival [8]. Furthermore, EOL PRT patients do not always benefit symptomatically [9], and treatment can occupy a significant proportion of the remaining life expectancy, which may be inconsistent with EOL goals [10]. Single-fraction RT (SFRT) is a viable alternative but is underutilised [11]. In fact, PRT in EOL treatment continues to be extensively studied; 5-10% of cancer patients of various kinds received PRT within the last 30 days of life [12]. In NSCLC patients, this figure is about 10% [13].

Given these considerations, our study aims to describe the patient and treatment characteristics of deceased NSCLC patients who received PRT, including the proportion of patients who received PRT during the last 30 days of their lives. We also aim to identify the prognostic factors to evaluate short-term survival in NSCLC patients treated with PRT. With these goals in mind, we hope to optimise palliative care for NSCLC patients and facilitate decision-making regarding PRT use in EOL situations.

## Materials and methods

Study design and setting

Our study was a retrospective study carried out at two RT centres within the Kent Oncology Centre (KOC): Maidstone and Tunbridge Wells NHS Trust and East Kent Hospitals University NHS Foundation Trust. Located in Kent, UK, the KOC provides cancer treatment to the entire Kent area, serving over 150,000 patients and performing more than 50,000 RT sessions annually. The study protocol was approved by the clinical audit unit for cancer service at Maidstone and Tunbridge Wells NHS Trust. The study covered two years, from January 1, 2022, to Jan 1, 2024.

Patient selection and data collection

Patient records were extracted from the main cancer patient database used in KOC based on specific selection criteria. Inclusion criteria included confirmed NSCLC patients who received PRT between January 1, 2022, and January 1, 2024, and deceased status before January 1, 2024, with a documented date of death. Exclusion criteria included patients under 18 years of age, those with recognised haematological malignancies, and individuals with an unconfirmed NSCLC diagnosis. Data collected included demographics, cancer diagnosis, histology, tumour, node, metastasis (TNM) staging, details of RT treatment (including sites, doses, and fractions), as well as recent chemotherapy or surgery within three months prior to the first PRT session. The Eastern Cooperative Oncology Group performance status (PS) and Adult Comorbidity Evaluation 27 (ACE-27) scores [14] were used to assess patients' PS and comorbidities, respectively, although they were not consistently available in the main databases. In cases where data were missing, oncologist and nurse medical notes were reviewed for PS estimation, while hospital admission and general practitioner records were used for estimating the ACE-27 scores. Information on recent hospitalisations within three months before the first PRT fraction was gathered from emergency department records.

Data analysis

The analysis used descriptive data to sum up patient characteristics in terms of categorical data as numbers and percentages and continuous variables as means. Categorical data have been tested using chi-squared tests, and constant variables using independent sample t-tests. Those who died over 30 days after the first PRT fraction (long-term survivors, LTS) and those who died within 30 days (short-term survivors, STS) have been compared to find significant differences. Multivariable logistic regression analyses were performed to determine potential predictors of receiving PRT within the last 30 days of patients' lives. Gender, age, PS, ACE-27 score, histology, TNM staging, recent hospitalisation, recent chemotherapy or surgery, completion of PRT treatment, RT treatment site and RT dose and fraction were considered as factors. All analyses were two-sided, and a p-value of less than 0.05 was considered significant. Data were statistically analysed using SPSS version 29 (IBM Corp., Armonk, NY).

## Results

Our study included 857 deceased patients who received PRT, of whom 709 (82.7%) survived more than 30 days after treatment initiation, and 148 (17.3%) died within 30 days.

Demographic and clinical features

In total, 418 (48.8%) of the 857 patients were female. Patients in the LTS group had a mean age of 70.9 years compared to 69.1 years in the STS group, though this difference was not statistically significant (p = 0.061). The two groups had similar gender distributions, with 45.9% females in the STS group and 49.4% in the LTS group (p = 0.449). The median survival time was 95 days for the LTS group and 19 days for the STS group. PS distribution varied significantly between groups (p = 0.027). PS 3 was identified in 52.0% of the STS group and PS 4 in 12.2%, compared to 44.4% and 6.8% in the LTS group, respectively. ACE-27 score distribution also differed significantly (p = 0.018). Notably, 12.8% of individuals in the STS group had an ACE-27 score of three compared to 6.2% in the LTS group. Within the STS group, recent hospitalisation was higher (35.1% vs. 31.3%) although not significantly so (p = 0.364). Detailed patient characteristics can be found in Table 1.

**Table 1:** Patient characteristics ACE-27: Adult Comorbidity Evaluation 27; LTS: long-term survival group; PS: performance status; STS: short-term survival group

Patient characteristics	LTS	%	STS	%	p
Gender	-	-	-	-	0.449
Female	350	49.4%	68	45.9%	-
Age (mean)	70.9	10.0%	69.1	46.7%	0.061
Survival time (days)	-	-	-	-	-
Minimum	31	-	2	-	-
Maximum	436	-	30	-	-
Median	95	-	19	-	-
PS	-	-	-	-	0.027
1	66	9.3%	12	8.1%	-
2	209	29.5%	30	20.3%	-
3	315	44.4%	77	52.0%	-
4	48	6.8%	18	12.2%	-
Unknown	71	10.0%	11	7.4%	-
ACE-27 score	-	-	-	-	0.018
0	172	24.3%	30	20.3%	-
1	292	41.2%	51	34.5%	-
2	137	19.3%	37	25.0%	-
3	44	6.2%	19	12.8%	-
Unknown	64	9.0%	11	7.4%	-
Recent hospitalisation	222	31.3%	52	35.1%	0.364

Disease characteristics

Within histology, the most common type of NSCLC in both groups was adenocarcinoma, with 52.2% in the LTS group and 55.4% in the STS group. The second most common was SCC, with 28.2% and 27.0% of the LTS group and STS group, respectively. Histological type distributions did not greatly differ between the two groups (p = 0.521). Regarding TNM staging, most patients had advanced disease. T4 disease affected 36.0% of the LTS group and 41.2% of the STS group for T stage. In both groups, N2 disease was very common (32.3% along with 35.1%, respectively) and N3 (22.7% along with 26.4%). Notably, M stage distribution differed significantly (p = 0.02), and M1 disease was represented by a greater proportion in the STS group (60.1% vs 47.5% in the LTS group). Detailed disease characteristics can be found in Table 2.

**Table 2:** Disease characteristics LTS: long-term survival group; SCC: squamous cell carcinoma; STS: short-term survival group

Disease characteristics	LTS	%	STS	%	p
Histology	-	-	-	-	0.521
Adenocarcinoma	370	52.2%	82	55.4%	-
SCC	200	28.2%	40	27.0%	-
Large cell	14	2.0%	0	0.0%	-
Other	125	17.6%	26	17.6%	-
T stage	-	-	-	-	0.183
0	4	0.6%	0	0.0%	-
1	64	9.0%	15	10.1%	-
2	114	16.1%	16	10.8%	-
3	108	15.2%	24	16.2%	-
4	255	36.0%	61	41.2%	-
Not staged	162	22.8%	32	21.6%	-
N stage	-	-	-	-	0.059
0	98	13.8%	17	11.5%	-
1	79	11.1%	9	6.1%	-
2	229	32.3%	52	35.1%	-
3	147	20.7%	39	26.4%	-
Not staged	156	22.0%	31	20.9%	-
M stage	-	-	-	-	0.02
0	203	28.6%	29	19.6%	-
1	337	47.5%	89	60.1%	-
Not staged	72	10.2%	20	13.5%	-

Treatment characteristics

Within the STS group, completion rates of treatments had been considerably less (63.5% vs. 82.8%, p = 0.001). The chest (47.3% in the STS group and 55.1% in the LTS group) had been the most frequent site of treatment, followed by the spine (25.0% and 22.3%, respectively). The average radiation dose (17.7 Gy vs. 19.6 Gy, p = 0.022), number of fractions (4.4 vs. 5.2, p = 0.019) and total dose (103.66 vs. 137.54, p = 0.009) were all significantly lower in the STS group. PRT regimens were generally 20 Gy in five fractions in both groups and were used to account for 55.4% (82/148) of the STS group and 49.8% (353 / 709) of the LTS group. No significant difference between the two cohorts was reported (p = 0.261); 33.1% (49 / 148) of the STS group and 36.8% (261 / 709) of the LTS group were treated with single fraction RT. No significant difference between the two cohorts was reported (p = 0.401). Fewer patients in the STS group had received recent chemotherapy (26.4% vs. 35.5%, p = 0.032). Recent surgery rates did not differ significantly between groups (6.8% vs. 8.9%, p = 0.399). Regarding concurrent treatments, chemotherapy was administered to 15.54% of the STS group compared to 16.50% of the LTS group (p = 0.027). Immunotherapy was used in 10.14% of STS patients and 12.55% of LTS patients (p = 0.62), while targeted therapy was employed in 2.70% and 6.06% of STS and LTS groups, respectively (p = 0.198). Detailed treatment characteristics can be found in Table 3.

**Table 3:** Treatment characteristics Gy: gray; LTS: long-term survival group; PRT: palliative radiotherapy; SFRT: single-fraction radiotherapy; STS: short-term survival group Recent treatment is defined as treatment within three months prior to the start of PRT.

Treatment characteristics	LTS	%	STS	%	p
Treatment site	-	-	-	-	0.427
Bone	64	9.00%	15	10.10%	-
Brain	59	8.30%	15	10.10%	-
Chest	391	55.10%	70	47.30%	-
Lymph node	13	1.80%	1	0.70%	-
Skin	3	0.40%	1	0.70%	-
Abdomen	12	1.70%	5	3.40%	-
Spine	158	22.30%	37	25.00%	-
Other	9	1.30%	4	2.70%	-
Dose	19.6	2.80%	17.7	12.00%	0.022
Fraction	5.2	-	4.4	-	0.019
Dose per fraction	5	-	5.2	-	0.269
3	147	20.70%	18	12.20%	-
4	350	49.40%	81	54.70%	-
Above 4	212	29.90%	49	33.10%	-
20 Gy in five fractions	353	49.80%	82	55.40%	0.261
SFRT	261	36.80%	49	33.10%	0.401
Recent treatment	-	-	-	-	-
Chemotherapy	252	35.50%	39	26.40%	0.032
Surgery	63	8.90%	10	6.80%	0.399
Concurrent treatment	-	-	-	-	-
Chemotherapy	117	16.50%	23	15.54%	0.027
Immunotherapy	89	12.55%	15	10.14%	0.62
Target therapy	43	6.06%	4	2.70%	0.198

Multivariable logistic regression analysis for early mortality risk factors

The analysis included factors of PS, ACE-27 score, metastasis status, treatment completion rate, radiation dose, number of fractions, and recent chemotherapy. Seven variables were assessed as predictors of 30-day mortality after adjustment. PS remained a significant predictor, with each unit increase related to 98.1% greater odds of 30-day mortality (adjusted OR: 1.981, 95% CI: 1.33-3.12, p = 0.001). Metastatic disease was associated with a 2.02 times higher risk of 30-day mortality (adjusted OR: 2.02, 95% CI: 1.246-3.571, p = 0.002). Patients who completed their PRT treatment had 66.3% reduced odds of 30-day mortality (adjusted OR: 0.337, 95% CI: 0.21-0.514, p < 0.001). Recent chemotherapy was associated with reduced odds of early mortality (adjusted OR: 0.542, 95% CI: 0.342-0.941, p = 0.044). Notably, several variables that were significant in univariate analysis lost statistical significance in the multivariable model. These were ACE-27 score of 3 (adjusted OR: 2.044, 95% CI: 0.93-4.17, p = 0.092), radiation dose (adjusted OR: 0.902, 95% CI: 0.674-1.311, p = 0.497), number of fractions (adjusted OR: 1.582, 95% CI: 0.579-4.791, p = 0.43) and concurrent chemotherapy (adjusted OR: 0.662, 95% CI: 0.435-1.007, p = 0.054). Detailed results for multivariable logistic regression are provided in Table 4.

**Table 4:** Results for multivariable logistic regression Recent chemotherapy: defined as chemotherapy within the last three months prior to the start of PRT, not necessarily on current chemotherapy Concurrent chemotherapy: receiving chemotherapy alongside with PRT ACE-27: Adult Comorbidity Evaluation 27 score; PRT: palliative radiotherapy; PS: performance status

Variable	OR	p-value	Adjusted OR	Upper 95% CI	Lower 95% CI	Adjusted p-value
PS	1.714	0.008	1.981	1.33	3.12	0.001
ACE-27 score of 3	2.332	0.012	2.044	0.93	4.17	0.092
Metastatic disease	1.849	0.008	2.02	1.246	3.571	0.002
Completion of PRT	0.365	<0.001	0.337	0.21	0.514	<0.001
Radiation dose	0.977	0.023	0.902	0.674	1.311	0.497
Number of fraction	0.94	0.019	1.582	0.579	4.791	0.43
Recent chemotherapy	0.649	0.033	0.542	0.342	0.941	0.044
Concurrent chemotherapy	0.532	0.027	0.662	0.435	1.007	0.054

## Discussion

Cancer treatment in the EOL setting is a complex and challenging issue, particularly when considering the role of PRT [15]. Although RT can often palliate symptoms, the effects are not immediate and are often accompanied by multiple clinic visits, which can be problematic in patients with limited life expectancy [16]. Data on PRT efficacy in the last weeks of life are limited, and studies report only modest symptom relief in a subset of patients [15]. This observation has led to a surge in interest in hypo-fractionated regimens [17], which provide optimal palliation with minimal treatment load. This complexity lies at the core of our work, which investigated PRT use and outcome in NSCLC patients at the end of life.

Our data suggest that 17.3% of our cohort died within 30 days of initiating PRT. This is significantly higher than reported previously [18]. In particular, earlier research shown that about 10% of patients with NSCLC underwent PRT in the last two weeks of life [13], while other literature indicates 8% in the last 30 days of life [19]. A systematic review shows that PRT utilisation during the last 30 days of life ranges from 5-10% in general cancer patients to 9-15.3% in PRT recipients [12]. The higher proportion observed in our study may be due to our patient selection, which included only deceased patients. Despite this methodological disparity, our data indicate that many patients are receiving PRT very close to the end of life, an observation that requires our attention.

It is important to note that some PRT indications, like spinal cord compression [20] or acute bleeding [21], may be considered critical even in patients with very limited life expectancy. While our analysis did not specifically examine differences in PRT indications between groups, the similar distribution of treatment sites across our cohort suggests that the indications for PRT were likely comparable among all patients.

A key finding is the low treatment completion rate in the STS group when compared to the LTS group (63.5% vs. 82.8%, p 0.001). This finding is in line with other literature findings [16,22], although our cohort exhibited slightly better overall completion rates. In fact, the potential benefit of PRT must be balanced with limited data on symptomatic relief in the EOL period. For example, studies have demonstrated that it takes three weeks to obtain pain relief in bone metastasis patients [17]. In another study, only 26% of patients reported palliation from RT during the last 30 days of life, and 52% had progressive complaints despite treatment [10]. These results highlight the potential for PRT to be ineffective or even harmful in some EOL cases.

Although we found that radiation dose, number of fractions and total dose were all significantly lower in the STS group than in LTS survivors, and only about one-third of the STS cohort received single-fraction RT (33.1%). This rate was not significantly different from that in the LTS group (36.8%). Although being underutilised, SFRT may provide the same palliative benefit with less burden in patients with very short life expectancy [23]. A single 8 Gy fraction can enhance quality of life and reduce pain in as little as 10 days and may be more suitable for very short life-span patients [24]. The benefits of SFRT go beyond its rapid onset of action; it also reduces the total treatment burden on patients through fewer clinic visits and generally fewer acute side effects [24].

Besides single-fraction RT, proton therapy emerges as a promising alternative for palliative care [25]. Proton therapy's superior dose distribution characteristics can potentially reduce side effects and adverse reactions in normal organs, which is particularly beneficial for lung cancer patients with poor PS, compromised cardiopulmonary function, and comorbidities, potentially helping to maintain or improve quality of life in these challenging cases [25].

The most common regimen in our cohorts was 20 Gy delivered in five fractions, representing 55.4% of treatments in the STS group and 49.8% in the LTS group. This differs from practices observed in other regions; for example, the most common PRT regimen in the US is 30 Gy delivered in 10 fractions [26]. Our findings suggest a trend toward shorter fractionation schedules compared to that of the US but still indicate a reluctance to widely adopt single-fraction approaches, even in patients with very limited life expectancy.

The underutilisation of SFRT that we observed is common and indicative of a general pattern in radiation oncology practice. Despite solid evidence from randomised trials demonstrating the efficacy of SFRT, several important issues may prevent many institutions from adopting this approach. Multiple-fraction regimens are widely used by radiation oncologists based on training and historical practice [27]. Our findings suggest a trend toward shorter fractionation schedules compared to that of the US but still indicate a reluctance to widely adopt single-fraction approaches, even in patients with very limited life expectancy. This could be due to the fear of complications; many oncologists believe that multifractionated regimens provide greater flexibility for managing these complex needs for PRT, but the evidence is limited [28].

The challenge is to estimate survival time in EOL conditions, especially when determining an appropriate PRT regimen or deciding whether to administer PRT at all. This difficulty often results in erroneous fractionation schedules that harm patients and result in poor treatment completion [10]. Our findings identified two significant predictors of short-term mortality that could aid in making these critical decisions, including poorer PS (adjusted OR: 1.997, p = 0.002) and metastatic disease (adjusted OR: 2.06, p = 0.005). This is consistent with other published prognostic models for patients with short-term survival time, including a model that identified certain cancer types (lung cancer, poor PS, advanced age, recent palliative chemotherapy, recent hospitalisation, and hepatic metastases) as predictors of worse short-term survival [29]; and another model with three factors including non-breast primary cancer, non-bone metastases, and poor PS were used to predict poor survival [30]. Although we did not investigate all these factors in detail, the focus on PS and metastatic disease is in line with these prognostic frameworks.

Interestingly, we observed lower odds of 30-day mortality with recent chemotherapy (adjusted OR: 0.542, p = 0.044). This finding contrasted with previous findings indicating that best - prognosed patients were more likely to receive aggressive treatment, such as palliative chemotherapy, despite questionable benefit and high side effects [18]. This could be due to a selection bias based on fitter patients are more likely to receive chemotherapy, or it could suggest a protective effect of recent chemotherapy in our specific patient population.

Several limitations of our analysis should be mentioned. Firstly, there were no clear data on the types of metastases, while some metastases, including hepatic metastases, are known to predict poorer outcomes according to previously mentioned survival models. Secondly, there was no PRT indication in the dataset, including the symptoms that required PRT in the first place, which limits the ability to interpret clinical reasoning behind treatment decisions. Thirdly, data were collected retrospectively and are potentially incomplete, especially for patients who were hospitalised or cared for in other trusts for which we hold no records. Lastly, because of the retrospective study design, we were lacking in symptomatic improvement or quality of life questionnaire data that would have evaluated PRT benefits in EOL patients.

## Conclusions

Survival time estimation in EOL conditions remains challenging when determining appropriate PRT regimens. Our results revealed poorer PS and metastatic disease as independent predictors of short-term mortality, highlighting the need for improved prognostic tools and better integration of prognostic information, especially considering the high use of multi-fraction RT within 30 days of death.
